# Balancing pandemic public health restrictions and family support at the end of life: palliative care and bereavement experiences of parents whose child died during the COVID-19 pandemic

**DOI:** 10.1186/s12904-023-01280-8

**Published:** 2023-10-27

**Authors:** Adam Rapoport, David B. Nicholas, Rosslynn T. Zulla

**Affiliations:** 1 Departments of Paediatrics and Family & Community Medicine, Faculty of Medicine, University of Toronto, Toronto, ON Canada; 2 Emily’s House Children’s Hospice, Toronto, ON Canada; 3 Faculty of Social Work, Central and Northern Alberta Region, University of Calgary, Edmonton, AB Canada

**Keywords:** Palliative care, Bereavement, Paediatrics, COVID-19, Care impacts

## Abstract

**Background:**

Little is known about the impact of the COVID-19 pandemic on families of children with chronic life-limiting conditions who died during the COVID-19 pandemic.

**Methods:**

In this qualitative study, parents of a child (< 18 years) who died during the COVID-19 pandemic from an underlying chronic medical condition were interviewed to explore how the pandemic impacted end-of-life care and bereavement experiences. Parents of children followed by the pediatric palliative care service were recruited from a large children’s hospital in eastern Canada.

**Results:**

Twenty bereaved parents, consisting of 12 mothers and 8 fathers, participated in individual interviews between January and December 2021. Findings identified impacts of the COVID-19 pandemic on children’s end-of-life care, experiences in hospital and at home, and family bereavement processes and experiences. Most parents experienced substantial worry about their child’s physical status and the additional risk of COVID-19 given her/his vulnerability. Parents also struggled to navigate public health protocols as they attended to their child’s needs and their family’s desire for engagement and support. Key facilitators that helped families cope included a strong network of formal and informal supports.

**Conclusion:**

Implications highlight the need to critically reflect on pandemic care in the context of co-occurring end-of-life processes. Findings amplify the need to balance necessary infection control practices with access to essential supports for families.

**Supplementary Information:**

The online version contains supplementary material available at 10.1186/s12904-023-01280-8.

## Background

The literature increasingly reports heightened strain on parents of children who required medically complex care during the COVID-19 pandemic. Strain reflected fear of the ill child contracting COVID-19^1^, a lack of resources to support parents’ information needs [1], additional family caregiver tasks [2], financial hardship [2], difficulty obtaining personal protective care equipment (PPE) [2], and challenges finding suitable childcare options for other children when attending to the needs of their ill child [2]. Furthermore, elevated stress was experienced by parents due to restrictive operational protocols in health care and community service delivery [2]. As an example, visiting policies restricting access of family members to be with the child in neonatal intensive care units reportedly resulted in negative psychosocial outcomes for parents (e.g., anger, sadness, a sense of not being able to protect their child) [3], relational challenges in families (e.g., less bonding between parents and child) [3], and strained communication between parents and service providers [4].

The implementation of public health protocols also altered how parents grieved the death of their child. Studies documented how in-person ceremonies (e.g., funerals, memorials) were delayed, restructured to virtual facilitation, or modified to comply with physical distancing and other public health protocols (e.g., decreased attendance, outdoor gatherings) [5, 6]. Among grieving parents whose child died of cancer prior to the COVID-19 pandemic, feelings of parental isolation due to physical separation along with stress due to changes in daily life (e.g., staying at home) were reported as the pandemic emerged and restrictions were imposed [5]. While some parents found that the changes in daily life and the subsequent isolation created opportunities to reflect on and process grief, others experienced strain due to feeling more distant from others [5]. A few parents also reported that behaviors associated with the pandemic (e.g., face mask wearing, hand sanitizer use) triggered negative emotions or memories of their child [5].

Despite emerging research indicating that the COVID-19 pandemic impacted the daily lives of families of vulnerable children, the experience of dying adults and their families, and the way that parents grieved the death of a child that had occurred prior to the pandemic, there is a paucity of studies documenting the experiences of families whose child received end-of-life care amidst the pandemic. To address this gap, this study explored the experiences of parents whose child died during the COVID-19 pandemic. Specific research questions were: (i) what was the experience of care and support during their child’s end of life?, and (ii) what was the impact of the pandemic on families after their child had died?

## Methods

Parents whose child died from their underlying medical illness (not due to COVID-19) during the pandemic were recruited from a large, tertiary care pediatric hospital in eastern Canada. Families were eligible if their child had been followed by the pediatric palliative care service, and study recruitment occurred between January and December, 2021. Consistent with other studies involving bereaved families [7], parents were approached at least 6 months after their child’s death out of respect for their recent loss, and to allow some time to pass so that parents might have some additional perspective. Eligible parents were sent information about the study by a member of the clinical care team who was not affiliated with the study. Information was sent by mail or email, and families were given the opportunity to opt out of further contact if they were uninterested and/or did not wish to participate. Families who did not opt-out were contacted after 2 weeks by a research team member (AR) to review the study invitation, answer questions, and provide assurances that participation was voluntary and that ongoing grief support would not be impacted by their decision. Parents who expressed a desire to participate were then connected with the study Coordinator, and an interview was scheduled. Interviews were conducted using telephone or videoconferencing technology (Zoom™) to allow physical distancing. Basic demographic data was collected about the parent(s) and the child, and the interview was guided by a semi-structured interview schedule. Questions explored how the COVID-19 pandemic impacted child and family access to services, and the family’s bereavement experience (Please see Supplementary File 1 for interview schedule). All interviews were audio-recorded and transcribed verbatim.

The interview schedule was developed by all three research team members (AR, DBN, RTZ). After trialing the interview schedule, it was modified to better elicit concepts and responses specific to parental experience of end of life and bereavement relative to the pandemic. Interviews were completed primarily by DBN, with the remainder conducted by RTZ. Both interviewers bring expertise in qualitative health research. To minimize role conflict, interviews were only conducted by team members who were not part of the clinical team and had no previous relationship with participants. Both interviewers reflected on their positionality and any potential biases that may emerge during data collection and analysis processes.

Transcripts were analyzed using a qualitative content analysis approach [8, 9]. This involved a three-step process of: (i) reviewing transcripts to generate units of meaning *(preparation)*, (ii) conducting an inductive analysis *(organizing)*, and (iii) developing a map that organizes the data *(reporting)*. The inductive analysis approach comprised three steps: generating units of meaningful data *(open coding)*, organizing these units *(creating categories)*, and developing themes. Rigor was demonstrated by data saturation, peer debriefing with clinicians, and referential adequacy to verify themes against raw data [9, 10]. Analysis was led by RTZ, with NVivo12 data management and analysis software being used to support the data analysis process.

The study protocol and informed consent procedure were reviewed and approved by the University of Calgary Conjoint Health Research Ethics Board (REB 20–0367), and SickKids Hospital Research Ethics Board (REB 1,000,070,092). Informed consent was obtained prior to study commencement, and participant privacy was assured, with identifying information removed from transcripts prior to analysis.

## Results

The parents of a total of 38 deceased children who met inclusion criteria were initially approached with an invitation to participate in the study. Of these, 20 parents were willing to be interviewed, comprising 12 mothers and 8 fathers. All parents had a child who had died during the COVID-19 pandemic, and each family had received support from the pediatric palliative care service in the hospital. All participants were proficient in English, with all communication and interviews conducted in English. Demographic characteristics of participating families are summarized in Table 1.

**Table 1:** Characteristics of participant families

	One parent interviewed	Two parents interviewed
Number of Interviewees	6	14 (7 dyads)
Ethnicity* (Number of Individuals Per Ethnicity)	African = 1 Canadian = 1 East-Indian = 1 Greek-Canadian = 1 Italian-Canadian = 1 Unknown = 1	British/Irish/Scottish = 3 Caucasian = 2 English/Irish/Scottish = 2 British/African = 1 Chinese-Canadian = 1 Eastern European = 1 European = 1 Guyanese = 1 Italian-Canadian = 1 Jewish-Kurdish = 1
Home Community	Urban = 3 Rural = 2 Unknown = 1	Urban = 10 Rural = 4
Number of Children in the Family	One Child = 2 Multiple Children = 3 Unknown = 1	One Child = 2 Multiple Children = 13
Child Characteristics		
Sex	Female = 3 Male = 3	Female = 2 Male = 5
Mean Age (Range)	8.4 years (6 months – 17 years)	5.8 years (< 1 month – 15 years)
Condition (Number of Children with Condition)	Hematologic = 3 Neoplasm = 2 Cardiovascular = 1	Neoplasm = 5 Genetic/Metabolic = 2

*As reported by respondents

Parents had mixed perceptions about the impact of the COVID-19 pandemic on their daily lives, including end of life and bereavement processes. For most parents, the COVID-19 pandemic imposed additive stressors on the already overwhelming journey of coping with their child’s last days and death. Prior to the child’s death, most parents worried about the child’s heightened risk of contracting COVID-19, and its potential impact given the child’s underlying vulnerability. Parents also noted shifts in care delivery in the hospital and community that often hindered how they could interact with, and care for, their child in her/his last days of life as well as engage with others in their grief. For several parents however, the impacts of the COVID-19 pandemic were viewed as inconsequential given the enormity of their loss and grief. And in some cases, specific elements of the restrictions were viewed as helpful by allowing more private time with the child.

Overall, themes emerged as follows: shifts in end-of-life care; rituals and bereavement processes; facilitators supporting bereavement; and lessons gained from this experience. Each of these broad areas of experience and outcomes, along with corroborating text quotes, are presented below.

### Shifts in end-of-life care during the pandemic: daily life during the child’s last days

**Experiences while seeking care in the hospital**. In several cases, parental experience of supporting their child in their dying had begun prior to the COVID-19 pandemic. In such cases, parents were already navigating the child’s illness and care, but they additionally had to learn how to manage that care in the context of the pandemic. The increased risk of their child contracting COVID-19 sparked worry about their child potentially becoming catastrophically ill and facing an earlier death. These fears often were heightened when their child was in hospital due to greater exposure to more people, many with communicable infections including COVID-19. Extra precautions were undertaken by several parents (e.g., extra cleaning, sterilizing bottles prior to breastfeeding). In some cases, strict pandemic protocols in the hospital (e.g., wearing masks, physical distancing) created a sense of greater protection and safety for parents.

Despite a heightened sense of safety preservation due to public health precautions and strict hospital guidelines, negative impacts of these measures were also described. Protocols limited how parents could engage with their child and family when in hospital, with several parents characterizing these operational protocols as restrictive, inflexible, cumbersome and inconsistently applied by health care staff or on various units. Frustrations reportedly were compounded by rigid application of strict protocols and ongoing COVID-19 testing of very ill children who necessarily frequented the hospital. For instance, a parent conveyed hospital screening protocols as excessive particularly for young children who were undergoing complex procedures, as noted below:

*There was the worry… with [the child] having to get swabbed every single time he had any kind of in-patient stay or even, you know, coming in for the day which happened quite frequently. With chemotherapy treatment, there’s a lot of fevers and things… [yet] he would have to be swabbed every time… the full COVID test…. So that was obviously really distressing for him and for us. I think he had twenty some swabs… so that’s pretty awful for an under-one year old to have to go through, and for us to have to watch. Among all of the other medical procedures that you have to witness and be there for, it just added another level of stress.*

Several parents stated that their child found mandatory PPE to be inconvenient and intrusive, particularly when required during treatment protocols, as one parent recalled:

*You know, he [the child] wore a visor and an N95 mask the entire time that he was in hospital, and as he became more and more consolidated in his lungs. That was difficult because he required oxygen therapy in the last month of his life. So, you know, masking and face shields, they were very constricting and that was difficult at times.*

Parents also felt that restrictive visiting protocols created distance, and in some cases, unnecessarily impeded physical and emotional support to their child and other family members. While telephone and online support was accessed, parents described how the separation became overwhelming, and in some cases unbearable, as family members received updates about the child’s health status often including life and death issues, away from the child and others in the family, as illustrated by the following parent:

*I received a lot of horrible news via Facetime or phone calls sitting in parking lots…. I watched [the child]… start his chemotherapy via Facetime because I couldn’t be there [due to visiting restrictions].’*

Restrictive policies hindered access to resources needed by families. For instance, the shutdown of “non-essential” hospital mental wellness programs early in the pandemic hindered access of family members to support services, especially of concern during periods of critical care and the child’s final days of life. Closure of specialized housing accommodations made it challenging for some non-local families to find lodging near the hospital. The closure of these supports was characterized as abrupt, and some critiqued the way in which shutdowns were communicated to families, as described by the following parent:

*We literally got… a letter from [the hospital] basically like, “Okay, no more visitors as of tomorrow”. The only positive thing about that is… a couple [of] nurses that we were close to… actually gave us a little bit of a heads up… like “Hey, I think that this might be happening in the next day or so”. But if we didn’t have that, it literally just would have been a letter on our door like, “No more visitors tomorrow”…. And that was huge because… [the child]… only had like seventeen days of living, right. So that was huge.*

### Home-based experiences

Care at home generally was a positive experience for most families as they were able to create an atmosphere of togetherness, quiet and “safety from the world”. Many parents described thought and diligence in implementing and maintaining safety such as limited (or no) visitors, interacting with those outside the household in creative ways such as communication through a window to avoid direct contact, necessary shopping being done solely by a designated person, cleaning on an ongoing basis, and extensive hand sanitizing. The home became a place of reprieve from the stresses of the pandemic, including the rigors of pandemic protocols in the hospital. More importantly, the home was viewed as a place of family togetherness in supporting one another and relishing time with the child, as exemplified below:

*We embraced the fact that we were home alone so we could be together. There’s no stresses of having to have company or all these things. We had to look at it like, you know, we were blessed with that time. I don’t think there’s much you can say, like you just have to follow the rules and stuff, right? You get your groceries, one person goes, like you know, but it also gave us the time that… we were a family, no choice, like no distractions.*

For families who spent their child’s last days at home, the home reportedly allowed for flexibility and togetherness. Parents described their child’s death at home as more fully honouring the family’s need for emotional support from loved ones, while also nurturing desired normalcy even in such a stressful time. For instance, parents were often able to ensure that extended family members and friends were able to be with the child in ways that likely would not have been possible if the child had been in hospital or hospice, as a result of the restrictions in place in both of these settings.

However, homecare services sometimes provided navigational and logistical barriers and challenges. For instance, several parents found it challenging to find needed equipment (e.g., age-appropriate hospital beds) and staff resources (e.g., physical therapists to come to the home). Despite these issues, parents who opted for their child to stay home during her/his last days of life stated that the challenges outweighed the stresses of pandemic protocols in hospital, as highlighted by a parent:

*We took her home right when they literally decided on ‘alternate parenting’ [only one parent permitted in hospital], and I don’t think I would have been able to handle that at all. I mean I was always/usually by myself, but it was because he [spouse] was working. But if we were in [hospital], I wouldn’t have allowed him [spouse] to go in by himself. I would have been like, “No, I’m going twenty-four seven to… [be with the child];… you have to stay at home”. Like, I wouldn’t have given up my time, no way.*

### Psychosocial impacts

Parents described a range of stresses as they navigated their child’s end-of-life care during the pandemic. They described substantial worry about their child’s health status and mental well-being exacerbated by infection risk and imposed precautions and visitation restrictions. Whether the child was in hospital or at home, her/his compromised physical health was often a primary concern for parents as they balanced safety with mental health and support needs. The restrictive protocols (e.g., social distancing) created a sense of uneasiness and worry for parents as they were concerned that their child and others in the family would miss out on essential emotional support, as highlighted by a parent:

*There’s a lot of anxiety because people supported our family and our child so much and then to be like, you know, “Uh no, you can’t come, you know, like I’m worried about the lockdown or, you know, I don’t want to get a fine or you know, like… [the child]…can’t get sick or we can’t get sick” …and then you’re looking at…[the child]…going, “all he [the child] wants is some normalcy for the last couple weeks of his life when the lockdown happened”.*

Parents also experienced deep concern over the toll of their child’s death on other family members (e.g., other parent[s], sibling[s], grandparent[s]), particularly given less opportunity to be together and support one another. Parents further observed that their caregiving duties significantly increased and were often more difficult to navigate during the pandemic. While many did not attribute reasons for the increased caregiving duties, one parent felt social distancing protocols prevented her from receiving assistance from family members. Another parent similarly felt that daily caregiving tasks imposed additional strain which was heightened by no or little hands-on support after the child’s death. A parent stated that the intensity of caregiving stress was overwhelming particularly when support was not available due to social distancing protocols:

*I would have my mom come and help us, but… she couldn’t come anymore so it’s literally me [alone] dealing with [care] because no one knew what was wrong with… [the child],… and it was a lot,… and I guess that, yes, it absolutely has to do with COVID.*

### Experiences following the child’s death

Following the child’s death, multiple areas of intensified challenge were described specifically as a result of the pandemic. For instance, parents identified a lack of guidance, and resulting uncertainty about how to discard equipment and medication following the child’s death. One parent described local organizations’ unwillingness to accept home care equipment (e.g., wheelchair, hospital bed) reportedly due to pandemic-related infection risk as described below:

*We went to bring back the wheelchair and they (the rental agency) didn’t want [it]… because of COVID and I’m like, “Well I’m not paying for this anymore”… so we left it on the step. I don’t know… how they work that out for renting that, I can’t even drop it off, like they don’t want it. We had a lot of equipment too and things, like… a hospital bed, that were purchased and I wanted to donate, [but]… people don’t want it… because of COVID.*

Parents described end-of-life ceremonies being modified or postponed due to public health restrictions. Protocols for funerals, rites, rituals and receptions were revised due to restrictions in social gathering; accordingly, public health protocols may have impacted how families commemorated and in some cases, grieved. Bereavement support to parents were further impacted, as highlighted by the following parent:

*In [our cultural] tradition,… when someone loses someone in their family, the community comes together and there are people who stay with the family to help them with housework, groceries, cooking, cleaning, and kind of cheering up the family and the person who lost the family member, like emotionally as well…. We didn’t have that option [due to the pandemic].*

Despite these restrictions on social gathering and resulting losses, parents described finding new ways to commemorate and celebrate their child’s life. Activities included online funerals, virtual grief support groups, and socially-distanced memorials (e.g., ceremony or commemoration at home, outdoor events). For some, technology was viewed as invaluable for facilitating emotional support, as noted by a parent:

*I started doing some groups… like ‘grieving’ in Zoom meetings with [hospital grief support staff]…. They were excellent. It was good to talk with other parents that are going through or have gone through the same thing that we had, made you feel a bit normal.*

Overall, mixed findings regarding online supports and services was conveyed. Despite appreciating increased access due to online capacity, a few parents stated that these formats could not replace being present in-person with others.

**Coping with the child’s death.** Means of coping with their child’s death amidst the pandemic varied amongst participants. For all parents, the grieving process was considerable and often overwhelming. Many felt that the pandemic substantially and negatively affected end-of-life processes such as time with their child, rites and rituals, and the receipt of support from others. Several parents described a feeling of isolation in not having others present as they navigated bereavement, yet in some cases, this allowed time to process their child’s death, as reflected by a parent:

*I got a lot of downtime at home with my husband and my family where I got time to just like realize what I had been doing to myself because it’s hard to… when you’re in a ‘go go’ mode, it’s hard to like switch out of the ‘go go’ mode.*

Some parents felt that the enormity and depth of pain in losing their child rendered the pandemic and its impact relatively inconsequential. For many, coping with their child’s death was conflated with pandemic conditions, as recalled by the following parent:

*I think if you talk to any bereaved parent, I think… it’s not easy to identify the process, you don’t even know you’re going through a process, but I remember feeling relieved when he passed away. I cried a lot, but once the crying ended, a weight had been lifted.…. I felt like, ‘okay, I no longer need to protect him from himself, and I don’t need to protect him from COVID, I don’t need to protect him’. Like you feel so powerless in the face of all these things happening and I think initially… it was COVID so there was so much else to do. I think under normal circumstances [after the child’s death], I would have taken off for a world tour or left the country somewhere really far away because… I didn’t want anybody’s sympathies.*

As parents returned to daily routines some time after the child’s death (e.g., returning to work, managing household tasks), they were again confronted with the prominence of pandemic protocols. Several parents felt that public health requirements became awkward and uncomfortable, and in some cases triggering, particularly when returning to the workplace, as highlighted by the following parent:

*From my experience at [the hospital] like with the whole PPE stuff…, I was traumatized by that in a certain way because then I had to go back to work… I had to get into myself wearing PPE [at work], and I feel like the experience from [the hospital left me] really traumatized, maybe because I had to try and like desensitize myself to going back to work and wearing the same PPE that our experience was at [hospital].*

### Support for end-of-life processes in the context of the pandemic

Support was an important resource that was perceived to help parents navigate and cope with their child’s last days and death. Parents listed multiple sources of support: (i) personal resolve, (ii) family, (iii) friends, (iv) hospital and social care networks or organizations, and (v) their community. Throughout their journey, parents characterized their child’s clinical/palliative care teams (e.g., doctors, nurses, grief support coordinators, counselors, etc.) as compassionate, available, non-judgmental, adaptive to the varying needs of individuals and families, and engaged in ongoing communication with the family. An area of team support that was particularly valued by parents was the management of service delivery/pandemic protocols in the child’s last days to ensure support to the child and family. Parents noted that their child’s health care providers often advocated for families, and worked with administrative leaders to shift rules and restrictions to optimize family time together at critical junctures. As an example, a parent commented:

*We were very blessed that they made a lot of exceptions for us. I guess the [hospital pediatric palliative care] team saw that our situation was different so they allowed [the patient’s sibling] to come in, which was amazing…. Usually they’re not allowing family members in or anything like that, but for him it was like, “no, he can get a pass anytime he wants. He can come and visit [the patient] because they were close”…. They were saying, you know, she wasn’t able to have her massage therapist come in, which made her really sad, but I fought really hard and the [palliative care] team worked with me and said like, “we need to get this kid her massage because she really needs it”, like her body was just tired of sitting in a bed for two and a half months, you know.*

Parents described benefit from the informational, emotional and tangible support offered by hospital team members. Virtual support provided by hospital and local community groups was valued by parents both during the child’s care and after death. This form of support was viewed as more accessible than it would have been if offered in-person, and importantly was viewed to lessen infection risk associated with COVID-19. A parent noted:

*I think [the hospital palliative care] team – because of COVID – ended up having a lot of online programs and online resources…. It’s just tough because it’s been a year since he passed away, but every month they send us a letter or a poem written by another parent, an experience of another parent. Normally I would have had to access these by going [a lengthy distance] to where [the hospital] is…. A lot of that travel was cut out and I think that helped me. So I think the online piece [helped]…. They do the memorial as well, every few months [for] the parents and families…. All those things I’m pretty sure I wouldn’t have done if it wasn’t online.*

Support from their extended family was viewed to be an important resource to help parents cope with their child’s last days and death which, in some cases, was heightened by shifted circumstances imposed by the pandemic. For instance, one parent was grateful for the extra caregiving support provided by her mother as her husband had extraordinary work demands due to the pandemic. Another parent appreciated the educational guidance provided by a neighbor who was working as a nurse during her son’s last days. Parents described family members (e.g., grandparents, aunts/uncles) providing emotional (e.g., listening to their fears), physical (e.g., assisting parent with the child’s daily needs such as hygiene and eating), logistical (e.g., providing accommodation while visiting the child) and respite (e.g., caring for the child while the parent rested or attended to other responsibilities) care. This presence of family varied relative to differing points of the pandemic, largely based on social distancing rules. As noted, some families found the lack of such support difficult when it couldn’t be offered, and valued it when pandemic protocols eased and allowed such gathering.

Some parents described their reliance on personal traits/skills and actions (e.g., learning to be resourceful and find information, practicing self-care, keeping busy) to help them cope. Some identified discovering personal abilities through the process, including resiliency, adaptability and an ability to be an advocate, as important not only in navigating care, but also in supporting other parents and families. For instance, a few parents shared their lived experience as a bereaved parent via technology (e.g., podcasts) to better educate health care providers in supporting families at/after end of life. Of note, almost all parents indicated a desire to contribute to this research to ultimately help other families in such circumstances. Friends and community support were described by several parents, which included others offering respite (e.g., assisting the parent with the child’s medical care) or emotional support (e.g., fundraising to create a memorial for the child), yet this support varied over the course of the pandemic and associated restrictions.

Despite support received and gratitude for that support, parents felt that potential overtures of support were blunted by pandemic restrictions. While virtual care options typically promoted accessibility, several parents observed that online delivery methods rendered support less accessible and meaningful. Issues with internet connectivity and a lack of comfort using technology were sometimes an obstacle. For some, online support was not utilized. Several parents stated that online loss and grief support needs to be particularly tailored to specific demographics, as highlighted by one parent who felt that the pandemic imposed challenging restrictions for some family members:

*When my parents were having a really hard time with… [the child]… passing,… they don’t connect over Zoom…. So while I did send them to a grief group... and it was online and they tried to go to one, it didn’t really resonate for them. I think even now, they haven’t completely come to terms with… [the child]… passing away or they still suffer from it. But they also don’t know what can be done when a person doesn’t connect online. I think it’s much easier for the younger generation.… They’re more comfortable learning on screen than in person.*

### Recommendations to improve end-of-life care

In moving forward, recommendations from parents largely focused on three broad areas: (i) operational protocols in a pandemic, (ii) targeted support to families, and (iii) communication facilitation with families. They recognized the need for consistent implementation of operational protocols during a pandemic (e.g., cleaning, screening, visitation), but also sought careful review and calibration of protocols relative to the needs of patients and families especially during critical moments at the end of life (e.g., allowing as many family members as possible to visit the dying child in hospital). They emphasized the need for clear and timely communication, with an emphasis on compassion and explicit guidance for parents, as exemplified by parents of a dying child who lived an extended distance from the hospital and needed to find accommodation because only one parent was allowed in hospital. The other parent struggled to find alternative spaces because hotels and other public spaces such as restaurants and coffee shops were closed. He stated:

*I would say logistically the best thing they [hospital] could have done is thought about [the fact] that… only one parent can come in. But you’ve got to remember there’s going to be a lot of communication, and if that parent is from out of town and comes to the hospital, [she/he] doesn’t know where to park or where to go or what to do. And [if parking underground], they have no cell phone signal or no way of communicating with anyone [when] underground, [and]…there’s no washrooms down there.*

Parents further emphasized the need for supports to be accessible, available and tailored to fit the needs of families particularly during critical times in care. Some amplified the importance of better incorporating family members, including siblings, in the grieving process – especially important to prioritize within additionally constrained circumstances such as during a pandemic. To that end, developing online options for support emerged as an important and potentially long-term learning from the pandemic.

## Discussion

It has been said that there is nothing worse than experiencing the death of a child. Our study sought to explore the specific and intertwining impacts of the pandemic for parents who lost their child during the pandemic. Questions addressed how the pandemic had a bearing on family experience. Could the monumental experience of losing a child be distilled relative to the co-existing and confounding impacts of the pandemic and its imposing restrictive care protocols? Were there positive experiences or lessons that emerged from losing one’s child and grieving during a pandemic? The answers to these questions offer implications for providing end-of-life care and grief support to this population in such constrained contexts, and provide us with lessons to consider as we continue to navigate through and beyond the COVID-19 pandemic, and in preparing for future pandemics.

Parents indeed indicated that the COVID-19 pandemic substantially impacted all aspects of their family’s experience. The impact was pervasive and included processes of end-of-life care, important rituals around death, grieving, and returning to work. These findings build on other studies that suggest public health protocols implemented during the pandemic substantially shifted pediatric palliative care as well as families’ processes of grief and loss [2–5]. In this study, public health protocols and their resulting impacts on caregiving and support had multiple effects on care within both hospital and home settings, but these shifts were differentially experienced. For most, these experiences added substantial stress that parents had to cope with, all while navigating the end-of-life process. While some parents found the virtual supports initiated during the pandemic to be adequate and even more convenient, for others, these modifications were less ideal (e.g., teleconference meetings to share updates about the child’s health status), and variably did not meet their needs. These findings highlight the critical role of targeting end of life and bereavement supports to the specific needs of individual family members, even during a pandemic. Careful consideration is needed regarding the format, frequency and logistics of support provision and importantly, how these features of support may need to be nuanced relative to families’ needs in the changing dynamic of a pandemic.

In a recent case study, Ellis and Lindley [11] described a national telehospice that provided virtual pediatric hospice clinical and wrap-around supports to families across Scotland. Implemented by a team of clinicians, information technologists and volunteers, the telehospice intervention tailored support to families’ emergent needs during the COVID-19 pandemic. Targeted to specific family members (e.g., siblings), support addressed emotional needs, social needs (e.g., connection with peers), and financial needs (e.g., access to supplementary income supports). As the pandemic progressed, new supports were added to augment clinical support. This type of creative and adaptive support, which considers the unique needs of the individual and family together with the additional challenges of losing a child during a pandemic, appears promising and informative. Parents in our study described how common challenges experienced by bereaved parents, such as discarding home care equipment after the death of a child and returning to work, were compounded by the pandemic, and they likely would have benefited from tailored support.

Taken as a whole, the findings of our study highlight tensions between the public health responses relative to population safety and the profoundly difficult and personal experience of death and grief. Many participating parents indicated that COVID-19 restrictions substantially compounded challenges of loss and grief by reducing physical access to the child and decreasing available supports. Yet others conveyed that some pandemic-related restrictions vicariously granted time with their child and privacy in grieving, while offering virtual supports that some were more likely to access. These results indicate that care and support at the end of life require resources *not* reflective of a *one-size-fits-all* approach, but rather, need to be nuanced to the needs of patients, parents and families [12]. Finding ways to optimally address these needs amidst widespread public health restrictions, such as a pandemic, no doubt is programmatically and systemically challenging, yet critically important. Such care likely requires case-by-case consideration. This suggests a careful balance in applying a responsive yet also flexible approach. This nuanced response invites critical consideration of the psycho-emotional, financial, sociocultural, navigational and logistical needs of a child and their family in the context of child and family-centered care.

The implementation of accessible wrap-around supports rely on strong coordination and collaboration between service providers across sectors of health care, palliative care, bereavement support and pandemic planning/response. For instance, coordination between hospitals and medical equipment suppliers in pandemic circumstances may be helpful in providing logistical support to safely access and dispose of home care equipment and medical supplies. Likewise, coordination between employers, health care providers and bereavement supports may be helpful in facilitating parents’ (or others’) navigation of bereavement support and workplace re-entry.

The ability to pivot service delivery within a pandemic relies on a strong infrastructure and ethical principles that support malleability in care reflective of pressing individual and family needs. In a United Kingdom-based study [13], emergency care providers highlighted management support and strong relational dynamics (e.g., ongoing and effective communication) as essential to reconfiguring care practices in the pandemic. Parents in our study notably appreciated overtures of kindness and advocacy during their child’s end-of-life care. Accordingly, pandemic restrictions must not preclude a context that enables empathy and compassion in care.

### Study limitations and research recommendations

This study elicited the experiences of parents receiving palliative care and end-of-life supports in only one Canadian jurisdiction. Future studies with broader samples are recommended, including families of varying ethnocultural and social demographics. Research examining individual, family, health care provider and system impacts over the course of a pandemic or other health emergency is suggested, given that participant experiences and needs shifted as the pandemic evolved. Such research is anticipated to offer further guidance on how to better respond to the needs of families receiving pediatric palliative care during and after a pandemic or other health emergency. Accordingly, further study is recommended in guiding care in the context of pandemic preparedness, response, and recovery, including support for co-existing bereavement *and* post-pandemic adjustment.

## Conclusion

This study has identified experiences and needs of parents and their families facing the death of a child in the context of the COVID-19 pandemic. Findings importantly amplify the requisite of targeted and comprehensive supports for family members that are reflective of careful assessment of the particular needs of each individual and family.

### Electronic supplementary material

Below is the link to the electronic supplementary material.

Supplementary Material 1

## Data Availability

A summary of datasets used and/or analyzed during the current study is available from the corresponding author and team on reasonable request.
