# Supplementary material for: Balancing pandemic public health restrictions and family support at the end of life: palliative care and bereavement experiences of parents whose child died during the COVID-19 pandemic
Source: BMC Palliat Care. 2023 Oct 27;22:162. doi: 10.1186/s12904-023-01280-8 (PMC10604781; doi:10.1186/s12904-023-01280-8)
Supplement: Supplementary file 1 — Supplementary Material 1 [file 12904_2023_1280_MOESM1_ESM.docx]

**Supplementary File: Parent Interview Schedule**

**For “Balancing pandemic public health restrictions and family support at the end of life: Palliative care and bereavement experiences of parents whose child died during the COVID-19 pandemic”**

**Preamble**

Relatively little is known about how a pandemic such as COVID-19 has uniquely affected end-of-life care for dying children, and the grieving process for families. In this study, we would like to learn how families experienced health care at the end of their child’s life during the COVID-19 pandemic, and how they feel the pandemic has impacted their bereavement process. Our hope is that this study will inform ways to improve services, and make care better for children and their families. We collected information from bereaved families after the SARS pandemic of 2003, and would like to further understand if and how what we learned from the SARS pandemic affected care during the COVID-19 pandemic.

**Interview Questions**

1. Before we get into things related to the pandemic, I wondered if you would be open to telling me a bit about your child? (*Note to interviewer: This may be more challenging for parents who only experienced life during the pandemic, so interviewer will need to be mindful of this*.)
2. In our earlier study, we found that a description about one’s child during their last days offered important information about how care was provided and experienced by the child and family. Focusing now on the time leading up to the end of [**child’s name**]’s life, how do you think the COVID-19 pandemic and any associated care changes put into place because of COVID impacted your child and family?

Probes:

- Do you think it impacted the location of care during this time (e.g., hospital, home, hospice)? Would you have preferred to be in a different location? If so, why?
- What about the people who were present in [**child’s name**]’s last days? Clearly restrictions impacted numbers of visitors, but do you think this had an effect on your family’s experience? How so?

1. At the time of your child’s passing, as well as the time after, how do you think the pandemic affected your/your family’s experience of grieving and memorializing [**child’s name**]?

1. Clearly a big focus of the healthcare system during the pandemic has been on safety and infection prevention. During [**child’s name**]’s end of life, do you think the healthcare providers found the right balance between being safe (e.g. properly following the COVID-19 guidelines with respect to wearing masks, social distancing, etc.) *and* providing compassionate care? And “care providers” can include a range of carers (for example, paramedics, doctors, nurses, social workers, etc.). Please explain.

**If yes**, can you describe how was this demonstrated, and its impact on you and your family?

**If no**, was there too much focus on safety (and not enough on providing compassionate care) or was there not enough focus on following safety protocols when providing compassionate care? Please explain.

1. As you reflect on your experience, what advice do you have for healthcare providers in terms of what would be helpful to future children and families receiving end-of-life care during a pandemic?

1. Is there any other information you would like to share that you think would be helpful for researchers and healthcare providers to determine how to improve care for children and families in a pandemic?

Thank you for your insights and willingness to share your perspectives. We know that sharing this information may not be easy. But please know this is very helpful as we work towards improving care for children and families in a pandemic.
